# A Qualitative Exploration of Post-Injury Challenges and the Potential Role of the PTSD Coach Mobile Application to Improve Recovery Among Acutely Injured Patients

**DOI:** 10.1002/jclp.70085

**Published:** 2026-01-06

**Authors:** Maria L. Pacella-LaBarbara, Megan Hamm, Neil Kenkre, Enzo G. Plaitano, Natalee Gallo, Harry Morford, Eric Kuhn, Brian P. Suffoletto

**Affiliations:** 1 Department of Emergency Medicine, University of Pittsburgh, Pittsburgh, Pennsylvania, USA; 2 Department of Medicine, Center for Research on Health Care, University of Pittsburgh, Pittsburgh, Pennsylvania, USA; 3 The Dartmouth Institute for Health Policy and Clinical Practice, Dartmouth College, Hanover, New Hampshire, USA; 4 Center for Technology and Behavioral Health, Dartmouth College, Hanover, New Hampshire, USA; 5 National Center for PTSD, Dissemination and Training Division, Palo Alto, California, USA; 6 Department of Psychiatry and Behavioral Sciences, School of Medicine, Stanford University, Stanford, California, USA; 7 Department of Emergency Medicine, Stanford University, Palo Alto, California, USA

**Keywords:** acute pain, digital health intervention, emergency department, posttraumatic stress, PTSD Coach

## Abstract

**Objective:**

Digital interventions designed to prevent development of chronic pain and comorbid mental health symptoms, specifically targeting the acute injury recovery period, are in the early stages of development. The evidence-based PTSD Coach mobile application is a free resource offering cognitive-behavioral interventions to self-manage posttraumatic symptoms; it is used widely in varied populations, and it is feasible and acceptable to injured Emergency Department patients. However, patients’ subjective experience and feedback regarding optimizing the app are lacking.

**Methods:**

We conducted a qualitative study in which we recruited and interviewed 18 acutely injured adult patients (5 men; 13 women) at-risk for persistent pain and psychological problems. Participants were instructed to use PTSD Coach for at least 1 week; during the interview, participants discussed post-injury needs and challenges, their experience with the app, and general perceptions of its suitability to address post-injury distress.

**Results:**

Favorable feedback centered on the variety of user-friendly tools to help manage symptoms, ability to increase awareness of symptoms and identify and cope with distressing reminders of the trauma, and on facilitating openness and linking to resources for mental health treatment. Suggestions for improvement included: increased personalization through app onboarding, text messages and other notifications to prompt use, having voice-overs to read content to participants, and linkages to in-person mental healthcare if needed.

**Conclusion:**

These findings support PTSD Coach as a potential self-management tool to prevent the chronicity of maladaptive psychological reactions to injury and highlight features that may improve its utility for this unique underserved population.

Physical injuries prompt roughly 38 million Emergency Department (ED) (Prevention 2022) visits in the United States (U.S.) annually, contributing to pain-related and psychological complaints that can interfere with recovery and functioning. Specifically, over a quarter of injured ED patients report elevated posttraumatic stress disorder symptoms (PTSS) within 1-year post-injury (Lowe et al. 2021) and chronic pain (CP) that persist for years (Rosenbloom et al. 2013). PTSS (i.e., intrusions, avoidance of trauma-related stimuli, altered trauma-related cognitions and feelings, trauma-related arousal or reactivity) and CP are highly comorbid (Asmundson et al. 2002; Beck and Hickling 2018) and share common cognitive, affective, and behavioral features (e.g., fear, avoidance; attentional biases, depression and reduce activity; anxiety sensitivity) that contribute to the development of and/or maintenance of each condition (Asmundson et al. 2002; Bosco et al. 2013; Liedl et al. 2010).

Based on the mutual maintenance, fear-avoidance, and shared vulnerability models, there are multiple pathways through which physical injury can lead to CP (see Figure 1): Acute pain sensations can impact CP directly, or indirectly through catastrophic appraisals such as pain catastrophizing (PC) (Andersen et al. 2016). PC reflects rumination, magnification, and helplessness and robustly predicts pain (Schütze et al. 2018; Ziadni et al. 2018). In the wake of injury, PC may continue to fuel fear and interpretations of pain-related sensations as threatening (Attal et al. 2014), and prevent active coping to overcome fear (Bosco et al. 2013). This cyclical process of fear, avoidance, and hypervigilance to such sensations, symptoms which are also central to PTSD, contributes to depression, reduced activity and ultimately CP (Crombez et al. 2012; Gatchel et al. 2016). Although an initial fear-avoidance response (e.g., remove one-self from unsafe stimuli) may be appropriate in the context of injury, its overgeneralization and persistence to safe stimuli is maladaptive and maintains symptoms of both conditions.

Injury also triggers acute emotional distress responses including fear and PTSS, which directly contribute to the development of PTSD at 1-month post-injury, and indirectly contribute to CP via pain-related processes such as PC (Asmundson et al. 2002; Beck and Hickling 2018; Pacella et al. 2018). Specifically, emotional distress is critical to shaping catastrophic thoughts (Andersen et al. 2016) (e.g., ruminating about the event; magnifying fear or feeling unsafe/edge even in safe places; feeling helpless or that they are not able to navigate challenges), and even minor injury is sufficient to cause PTSS (Lowe et al. 2021), which in turn can prompt and maintain PC (Asmundson et al. 2002; Beck and Hickling 2018; Pacella et al. 2018). Notably, among ED patients with mild-to-moderate motor-vehicle crash (MVC)-related injuries, PTSS assessed in the ED and 1-month later uniquely contributed to PC at 3-months, above and beyond demographic (e.g., race and sex), injury-related (initial pain score), and psychological characteristics (initial PC score, preexisting PTSS) (Pacella-LaBarbara et al. 2023b). Directly addressing these malleable psychological factors may optimize recovery and contribute to the national priority of CP prevention (Finstad et al. 2021; Martin et al. 2021).

The acute post-injury period (i.e., within 3-months post-injury) is a unique time for intervention to improve psychological response and recovery. Cognitive-behavioral therapy (CBT)-based treatments are successful for reducing CP and PTSD, and also show promise for acute PTSS and depression among high-risk patients (Pacella-LaBarbara et al. 2023a) when delivered within the acute post-injury period. However, traditional CBT is a time- and resource-intensive treatment that is commonly offered in-person by highly trained providers, limiting availability and uptake after injury. Further, several patient-related barriers limit engagement in traditional mental health treatments immediately post-injury, including stigma (Aaron et al. 2024; Ruzek and Yeager 2019), lack of access to or knowledge about mental health, concerns about confronting trauma-related symptoms (Kantor et al. 2017), lack of time, interest and/or transportation, and other social, financial, and logistical health-related challenges (Aaron et al. 2024; Kantor et al. 2017; Price et al. 2018). Moreover, there is a gap in the availability of psychological services to address acute symptoms with limited integration of mental health care in traditional medical settings (Aaron et al. 2024), no standard of care for identifying patients in need, and no standard early intervention (Pacella-LaBarbara et al. 2023a; Wong et al. 2009). These barriers are problematic in the ED for those with mild to moderate injury who are discharged home with few or no resources (Pacella-LaBarbara et al. 2023a).

PTSD Coach is a free and publicly available self-management mobile application (app) developed by the U.S. Veteran Affairs National Center for PTSD; as a digital mental health intervention, it is well-suited to overcome many of these barriers by providing private and easily accessible tools and resources for distress self-management in the acute post-injury period. PTSD Coach is both feasible and acceptable to injured ED patients (Pacella-LaBarbara et al. 2020), and the app is a popular resource both in the U.S. and globally (Kuhn et al. 2018). Given the overlap in CBT-approaches for PTSD and pain, PTSD Coach is already equipped with components of CBT for CP (CBT-CP) that are used to address psychological contributors to pain (e.g., PC; See Figure 2) (Bosco et al. 2013; Murphy et al. 2022). This CBT-based content can be accessed in real time within four primary modules of the app: (1) “Learn” presents psychoeducational materials about PTSD, its management, and its overall impact; (2) “Track Progress” allows for symptom monitoring via PTSD assessments; (3) “Manage Symptoms” includes over 20 CBT-based tools (see Figure 2) designed to address both PTSS and general reactions to trauma, such as feeling disconnected from people, depressive and anxiety symptoms, etc. These tools are targeted at restructuring maladaptive appraisals and cognitions, identifying and coping with triggers, promoting active coping and emotion regulation (e.g., mindfulness), using behavioral activation to improve mood (e.g., meaningful activities; socialization), and enacting problem-solving skills (Bosco et al. 2013). (4) “Get Support” provides resources regarding crises, professional care, and growing your own support network. PTSD Coach was perceived as acceptable and moderately-to-very helpful for managing PTSS among Veterans (Kuhn et al. 2014) and was shown to reduce PTSS among civilians in individual trials (Hensler et al. 2022; Kuhn et al. 2017; Miner et al. 2016). PTSS, depression, and psychosocial functioning have improved in civilians after 3-months of use (Kuhn et al. 2017). However, only one pilot study has examined the potential usefulness of PTSD Coach as a prevention and/or self-management tool among acutely injured ED patients without evidence of a PTSD diagnosis or elevated PTSS (Pacella-LaBarbara et al. 2020). Although the primary trial did not yield a significant benefit for the PTSD Coach app, secondary analyses revealed a 7-point lower PC score among PTSD Coach participants compared to those in the control group, and Black participants with high levels of PTSS at baseline reported marginally significant but robust improvements in post-injury PTSS and significant improvement in PTSS coping self-efficacy (Pacella-LaBarbara et al. 2020). Given that the PTSD Coach app has traditionally been used for patients with established PTSD symptoms, its use as a prevention tool is novel, and further research is needed to determine whether PTSD Coach is suitable for the needs of acutely injured patients.

## 1 | Current Study

Whereas it unlikely for a stand-alone digital mental health intervention to mitigate maladaptive cognitive-behavioral strategies that have become entrenched over months or years (Hensler et al. 2022), there is high potential for such an approach to foster healthy behaviors (e.g., healthy sleep habits; enjoyable activities; changing negative thinking patterns; improving problem solving skills and relaxation techniques, etc.) that may prevent chronicity of maladaptive patterns, reduce negative emotions, and improve quality of life. Digital mental health apps, like PTSD Coach, are often considered low risk, given their potential to offer timely, low cost, and discreet mechanisms for patients to self-manage their mental health symptoms (Ha and Kim 2020; Kretzschmar et al. 2019; Marshall et al. 2020). Importantly, patients can access resources both anonymously and remotely with less risk for negative social perceptions and stigma often reported during face-to-face psychotherapy (Ha and Kim 2020; Marshall et al. 2020; Price et al. 2014). To that end, PTSD Coach is a low-risk intervention equipped with CBT-based content to target psychological responses that may reduce post-injury PTSS and PC. Yet, no user experience data exists from patients in the acute post-injury period to understand its suitability to address their unique needs.

Most qualitative feedback about the PTSD Coach app has been limited to Veterans with chronic PTSD (Kuhn et al. 2014), and highlight favorable features such as the ability of the user-friendly platform to: (1) equip users with customizable resources on their phone to help understand and explain symptoms; (2) monitor symptoms and manage acute distress, including PTSS and sleep problems (Harned et al. 2023); and (3) augment and become more engaged in traditional face-to-face therapy (Possemato et al. 2017; Shakespeare-Finch et al. 2020). Mental health clinicians also view the app favorably, citing its ability to facilitate symptom monitoring engagement in care and participation during and between in-person sessions, and offer different coping strategies between clinic visits (Possemato et al. 2017; Strodl et al. 2020). Barriers to app use included technical issues and complaints about outdated content and insufficient resources to cope with symptoms (Harned et al. 2023; Possemato et al. 2017; Shakespeare-Finch et al. 2020). Notably, severity of symptoms, both in the direction of those with minimal (e.g., no need to use it) and severe symptoms (e.g., resources are insufficient) (Corthésy-Blondin et al. 2023) may limit engagement with trauma-related apps in general.

To address the gap in qualitative work among civilians with acute injury, we conducted semi-structured in-depth interviews among adults with recent physical injury that prompted an ED visit. Based on prior work (Corthésy-Blondin et al. 2023), we targeted those at high risk for developing PTSD. Although most studies reported here involved patients who sustained a MVC-related injury as the primary mechanism, many studies also include mixed injury samples thereby representing the general injury population; for this study, we therefore targeted a mixed injury sample. Interview prompts were designed to elicit feedback for three overarching study goals, to: (1) identify the unique emotional and physical health needs and challenges that emerge after injury and understand management of those needs; (2) determine whether PTSD Coach could help address those needs to improve post-injury recovery; and (3) obtain feedback about ways to optimize the app to reflect their unique needs and enhance app engagement.

## 2 | Methods

The University of Pittsburgh IRB approved this study (STUDY21060019). We applied a Qualitative Description approach, a created in the field of Nursing to describe the thoughts and experiences of participants’ on study topics, and common in qualitative studies conducted in Medicine and the Health Sciences (Kim et al. 2017; Sandelowski 2000). Qualitative interviewing and analysis were handled by Qualitative, Evaluation & Stakeholder Engagement Research Services (Qual EASE), a research core at the University of Pittsburgh that employs, trains, and supervises full-time qualitative research specialists. This research study utilized private one-on-one interviews between a trained researcher and participant for data collection about the PTSD Coach app (https://mobile.va.gov/app/ptsd-coach). This study design was not preregistered; transcripts are not available for reasons of privacy, particularly given that we did not include a data sharing plan in the original Institutional Review Board submission of this protocol, and the study is now closed for modification; yet, interested researchers may contact the study team for collaboration and codebook information. Please see the supplemental material for the interview guide. Descriptive data were analyzed using SPSS version 28 (Corporation 2021) and qualitative data were analyzed via MAXQDA 2022 (Software 2022).

### 2.1 | Participants

We recruited 44 adults (ages 18–65) who sought ED treatment for a physical injury that occurred within 90-days prior (e.g., motor vehicle crash, interpersonal trauma-fight, sports, fall). Additional eligibility criteria included English-speaking patients who owned a smartphone with the ability to download apps, self-reported serious injury and/or life threat from the index injury (i.e. Criterion A of the PTSD diagnosis), endorsed a score of ≥ 15 on the Posttraumatic Adjustment Scale (O’Donnell et al. 2008) indicating risk for PTSD stemming from the index injury, were discharged directly home from the ED (e.g., injuries unlikely to warrant hospital admission), and had a pain score ≥ 4 (out of 10) on the Numeric Pain Rating Scale. Exclusions included pregnancy, and sexual assault or abuse given that these groups may benefit from additional resources for complex post-injury challenges that are not included in PTSD Coach. Additionally, given low mHealth usage among older adults and barriers associated with mHealth in this population (Li et al. 2021; Pywell et al. 2020), we excluded people over the age of 65.

### 2.2 | Procedure

The Human Research Protection Office of the University of Pittsburgh (Study 21060019) approved all study procedures. Using convenience sampling, participants were primarily recruited in-person directly from the EDs of two Level I trauma centers in Pittsburgh, Pennsylvania. Two additional recruitment methods included advertising via flyers posted in the EDs of the two trauma centers and via the University of Pittsburgh online research registry.

For in-person recruitment, research assistants (RAs) identified potential participants through FirstNet, a platform used to track ED patients and assist with clinical functions, then approached the treating team to confirm eligibility. An RA then approached all potentially eligible patients in the ED, briefly described the study to those interested, and further confirmed eligibility through screening prior to obtaining verbal consent. Screening was completed on an iPad via the Research Electronic Data Capture (REDCap) website (Harris et al. 2019).

For recruitment via the flyer method, interested patients reached out to the study team and were screened for eligibility via telephone. Similarly, interested participants recruited through Pitt+Me viewed an online advertisement and completed initial screening through the website; if they were eligible, they were contacted by an RA to conduct the full screening assessment via telephone. All participants provided verbal consent for study procedures.

### 2.3 | The PTSD Coach App

Following consent, an RA assisted participants with downloading PTSD Coach onto their phones (App Store or Google Play marketplaces) and provided them with a brief (5-min) introductory tutorial on how to use each of the 4 app modules: (1) Learn, (2) Track Progress, (3) Manage Symptoms, and (4) Get Support. The Learn module provides basic information about PTSD, how it can affect people, and an overview of possible treatment options. In the Track Progress module, users have the option to complete the 20-item PTSD Checklist - 5 assessment (PCL-5), track and receive feedback on their scores over time, and schedule future assessments. The Manage Symptoms module includes tools for common PTSD symptoms (e.g., avoiding triggers, disconnected from people) and provides customized tools (e.g., deep breathing, grounding techniques) for them. Lastly, the Get Support module provides information regarding emergency and crisis support, finding a therapist in the participant’s area, and connecting with peer-support. All participants were instructed to use and explore the app at their convenience over the next week.

### 2.4 | Interview Process

All interviews were conducted remotely via Zoom by a trained interviewer from Qual EASE (the interview script can be found in S1 material). Following enrollment, the interviewer contacted participants to schedule a private interview occurring at least 1 week after enrollment. Participants received a $30 amazon gift card for interview completion.

### 2.5 | Measures

We recorded participant demographics, the mechanism and date of injury, and current receipt of mental health therapy and/or counseling for a psychiatric disorder (yes/no).

## 3 | Data Analysis

### 3.1 | Sample Demographics

Of 214 patients approached in the ED, most (*n* = 146; 68%) were screened for eligibility; 32% (*n* = 68) declined, primarily due to lack of interest (*n* = 43; 59%), time (*n* = 15; 23%), or other reasons (*n* = 10; 18%). Screening status could not be determined for 3 patients due to a refusal (*n* = 1), syncopal episode (*n* = 1), and an unknown medical reason prior to completion of the screening assessment (*n* = 1). Of the remaining 143 screened, 30% were eligible (*n* = 41) and 70% (*n* = 102) did not meet inclusion criteria, primarily due to lack of Criterion A trauma exposure (48%; *n* = 49) and a low score (< 15) on the Posttraumatic Adjustment Scale (PAS), indicating low risk for PTSD (33%; *n* = 34). One of the eligible participants was deemed inappropriate as they were admitted to the hospital for observation shortly following screening; as such, 40 participants were recruited via in-person methods from the ED, and 15 completed their interview. An additional 6 patients were screened for the study via the research portal (3% of the full sample); of these, 4 were ineligible due to a low PAS score (*n* = 2), lack of Criterion A trauma exposure (*n* = 1), and injury > 90 days ago (*n* = 1). The remaining 2 were eligible, enrolled, and completed the interview. Additionally, 2 patients were screened after viewing the study flyers in the ED (1%); both were eligible and enrolled, yet they did not complete the interview.

In total, 44 participants (27 females) provided verbal consent; participants were screened an average of 2 days (SD = 5.85 days) after their index injury, with 74% of participants being screened either on the same day (*n* = 98; 63%) or within 1 day of injury (*n* = 16; 11%).

Despite multiple attempts to contact participants (via phone calls, text messages and emails), only 18 (41%) completed an interview: 20% (*n* = 9) did not appear for their scheduled interview, 32% (*n* = 14) were lost to follow-up (some phone numbers out of service) and/or their eligibility expired prior to scheduling an interview, and 7% (*n* = 3) withdrew due to lack of time, a family emergency, and hanging up prior to beginning the interview. Women (52%) were more likely to complete an interview compared to men (23%); (χ^2^ (1) = 3.46; *p* = 0.06) (non-significant trend).

The 18 participants who completed the interview were primarily non-Hispanic (94%; *n* = 17) women (78%; *n* = 14), averaging 40 years old (SD = 13.21; range 18–62). Participants primarily identified as White (61%, *n* = 11), followed by Black (28%, *n* = 5), multiracial (5.5%, *n* = 1), and mixed race (5.5%, *n* = 1). Falls were the primary mechanism of injury (61%), followed by general accident (17%, *n* = 3), motor vehicle (11%, *n* = 2) and motorcycle crash (5.5%, *n* = 1), and interpersonal trauma (5.5%, *n* = 1). Participants reported high pain (*M* = 8.61; SD = 1.61), and nearly all (89%) reported that they sustained or could have sustained a serious injury; 50% endorsed life threat. Over a quarter (27.8%) had active mental health therapy or counseling.

### 3.2 | Qualitative Interview Summary

All interviews were conducted in the zoom platform; transcripts were automatically generated by zoom then manually corrected and produced (verbatim) by trained transcriptionists; identifying details were redacted. The average length of interviews was 23 min, though they varied in length from 12 to 36 min, with the degree to which the participants had used the app, and natural variation in their talkativeness, accounting for such variation. Transcripts ranged from 1,952 words to 6,658 words, with a total word count across all transcripts of 62,772 words.

### 3.3 | Qualitative Interview Analysis

Under the supervision of the qualitative methodologist (MH), trained, experienced analysts at Qual EASE inductively developed a codebook reflecting the content of the interviews. Coding was facilitated by MAXQDA22 Software. Use of the codebook was practiced on two transcripts by 2 Qual EASE coders, following which they both applied the codebook to 10 transcripts. Kappa statistics were used to assess intercoder reliability; the average kappa score was 0.77, indicating “substantial” agreement. The two coders met to fully adjudicate all coding discrepancies, following which the primary coder independently coded the remaining 6 transcripts. The primary coder for the project then conducted conventional content (Hsieh and Shannon 2005) and thematic analyses according to the steps outlined by Braun and Clarke (2006) (Clarke and Braun 2021), which was reviewed by the qualitative methodologist, and shared with the study team for review, discussion, and refinement.

## 4 | Results

Through thematic analysis of the interview data, we identified six themes, which are described below in turn. Although we targeted patients not requiring hospitalization, we did not have access to medical records to confirm discharge to home. As such, it became evident during interviews that some patients were hospitalized following enrollment, unbeknownst to the RA, or may have been recruited during an ED visit prompted by re-injury from prior hospitalization.

### 4.1 | Theme 1

#### 4.1.1 | Post-Injury Pain and Stress on the Path to Recovery

Participants describe significant challenges with post-injury pain, though the severity of pain varied by individual and type of injury. Even those who were accustomed to dealing with pain were surprised by the types of pain associated with new injuries. One participant described how the pain from her broken fibula was much worse than the pain from a previously severely broken wrist: *Yeah, it was horrific [Laugh]. And I’ve broken, like, I broke my wrist before in four places and this is way worse…*. Participants generally described having good pain control while in the hospital, but noted that pain often worsened as the shock of the injury wore off, or as recovery progressed—and that medications that they were discharged with were not sufficient for pain management:

Tylenol and Motrin together’s all grand, but it’s not really super effective for broken bones…

Many participants also experienced significant stress from disrupted work, family, and social routines that they had to manage in addition to their injury-related pain and recovery. For example, one participant was unclear on his employment status because of delays in the hospital transmitting necessary paperwork to his employer explaining his injury:

Honestly, the biggest stress I have right now is my job. […] They were supposed to hold it for me and after six6 weeks my doctor was supposed to submit some kind of form. Well, I can’t make my doctor submit a form; all I can do is call the office. […] I’ve had several threats against my job because they didn’t turn in paperwork on time. […] I guess that’s just my biggest stress right now is me not having that job anymore. You know, I got eight kids total; I got five little ones under, under the age of thirteen.

Yet another participant described her limited mobility and consequent impact on activities:

Um, I recently slipped on a patch of black ice. I have two herniated discs in my back, and I…kind of pulled muscles, and I think there’s a pinched nerve in there. I physically can’t really move my left hip cuz of the way I fell.[…]. Like, I recently had to borrow a wheelchair because we did a lot of grocery shopping this weekend and I just couldn’t stand.

### 4.2 | Theme 2

#### 4.2.1 | Participants Also Faced a Range of Logistical, Informational, and Existential Challenges on the Path to Recovery

Specifically, logistical challenges included navigating healthcare or insurance paperwork and challenges related to transportation. For one participant, these logistical challenges compounded one another:

You know, the stressful part of it is that my car is not drivable, I’m not able to return to work, so there’s like the financial concern. There’s the stress of, I have a twenty-year-old who’s pregnant—so, you know, there’s like all of these outside factors that are now influenced, kind of negatively impacted by this accident that was just like… […]So, not so much with the pain, but the frustrating part about it that is like not feeling well, not feeling 100% myself, and then having to manage like, the paperwork. It’s not easy to go through work and apply for like short-term disability, which I qualify for, it’s just like, you know it’s a hassle.

Informational challenges added further layers of stress with participants struggling to navigate the healthcare system for information about their condition and recovery:

I feel like in the hospital, it’s kind of one thing, you have someone who can kind of seek out answers for you, or they have more immediate access to a solution or different types of solutions. And then, I feel like being home is like, a whole other like scenario, right? Like it’s a matter of trying to message through like [health system] app or contacting the doctor, or just, you know, straight up using Google to try to find some sort of relief for pain management or just solutions to some of those things… and they’re not always—you know, if you’re not using critical thinking skills, they’re not always the most accurate, it’s not out there on the internet as the most accurate information. So, the time-management wasn’t a concern in the hospital, but at home it’s kind of like, I’m feeling this now, I don’t want to wait 6 h or 3 days to get an appointment to see you for, and I don’t want to use the ER as my PCP, which I think is ridiculous.

For some participants, there were also existential challenges related to managing uncertainty, and to having to rely upon others, an uncomfortable position for independent and reliant individuals:

I’m a very, very, very independent woman. I do everything by myself; I always have and I always will. Physically counting on someone or depending on someone else is very hard for me, uh, because I’ve just always been just me. So, when I have to have someone help me to go to the bathroom or down the stairs, and they don’t, like, get there, I’m gonna try to do it myself, and if I get hurt that’s on me, because I know I should wait and I know I should take my time. […} So, now that I just can’t[…do it myself], it’s a struggle, it’s stressful, it gives me anxiety, it puts my mental health at risks…it’s a lot.

Those who did not struggle with depending on others might still struggle with the overall uncertainty of their situation. When asked what was most stressful for him, one participant said that it was *Just the timeframe. I don’t know how long it’s gonna take…*

### 4.3 | Theme 3

#### 4.3.1 | PTSD Coach Helped Participants to Manage Stress and the Existential Challenges of Their Injuries

Participants who actively used the app (*n* = 12; 66%) described it as helpful in managing stress and the existential challenges of injury. Several participants mentioned that the app contained tools with which they were familiar from past experiences in therapy, including PTSD treatment. What they particularly liked about the app was having everything included in one place, as in the following description:

So, I think it’s similar, um, as far as…tools that I’ve used, but I feel like there’re more, all, like there’re a lot of them all on one app, versus if I’m maybe just like, okay, I’m anxious and I go on something else, it might not have as much information, if that makes sense. So, this has where you’re kind of having a lot of different tools that you can utilize in the same app, so I like that about it. And that you can kind of just pick, like, okay, so what’s wrong? What am I doing? Am I sad, am I feeling disconnected, am I, you know, whatever; versus just, okay, I’ll throw this meditation on cuz this is for anxiety.

The tools themselves, particularly in the Manage Symptoms section, helped participants to navigate negative emotions and distress. One participant describes how the app helped her to break the cycle of rumination, a maladaptive feature central to both PTSD and PC:

Yeah, that is. And those, I think, are really helpful, um… because it gives you an opportunity just to take a step back and, …and separate yourself from your, uh, cycling, stressful thoughts […]; that’s one of the things that an anxious person does is your thoughts just keep — you know, you don’t have the ability to just cut off the thought. Like, alright, I’ve done that, I’m putting it away for now; you, you have a hard time putting it away in the box, so, and then, so that you can take it out later when your mind is, you’re more relaxed and ready to handle that thought. It just stays in your head. So, I mean the exercises is helpful in being able to put the thought away[…. To help stop the cycle].

One participant described using the app to help deal with feelings of social isolation, stemming from her immobility and stress over an exam she had been preparing for. During her recovery and struggles, she discovered that the app was a perfect match for easing her emotional pain:

At first it was the pain, my, the pain that I felt in my body, but later it, it became the pain of the surrounding environment. When I went to the hospital, and then I received medication, I realized that it wasn’t that bad. But then, sitting at home just being in such an impediment… it got me to an emotional part of the pain, that I feel like I was lonely. I was feeling like I cannot do most of the things that I’ve been doing. I just felt left out; I feel like I was losing on my life… At first, when I downloaded it, I thought it maybe a manual for maybe managing your physical pain… But when I downloaded it, I realized it was something that could just treat your physical pain but also your emotional pain. It was really helping; I think it’s just perfect…. Over the last week…. I used it to track my progress on Monday. But every day I, I open it just for my encouragement?

Other participants similarly mentioned that relaxation and distraction techniques were helpful to cope with stress and pain, and that the app proactively re-oriented them towards thinking about things that bring them joy. Another participant experienced considerable stress after losing her vehicle and reliving the accident. The app helped her understand her triggers, lower distress, and find comfort in the knowledge that she attempted something to make herself feel better:

I’ve dealt with a lot of stress over losing my car and I get stress whenever I think about the situation in general, because I was very, very terrified when it happened. Uh, I had used the app and I’ve talked to my therapist… I actually tried using, like, the app for, like…warnings or, like, avoid, like, triggers and stuff like that…. I do like the app to, like, answer questions about PTSD, and how multiple symptoms that it helps you with… In general, I still feel pretty good knowing that I’ve tried something.

Similarly, another participant described how the Manage Symptoms section helped her identify and cope with triggers:

I really like the managing my symptoms, because like, the more quickly I identify what I’m actually feeling, like the easier it is to cope with it. So like, I kind of really like that that section the most, because it gave me, you know additional information and things to manage what I’m going through, because sometimes I think I’m feeling one way, and it’s really a reaction of the PTSD, you know what I mean? And, like the nightmares and stuff too, like you know, when I see stress coming like, you know, I can manage my expectations, so I A, don’t get frustrated with myself, and B, I’m more like self-aware, so when it does happen I’m not as like all over the place.

[laugh]

In sum, participants valued the aspects of the app that help them learn about their symptoms and identify and regulate their emotions; certain features in PTSD Coach may be helpful in differentiating between their thoughts and feelings, as well as between generalized anxiety versus post-incident acute stress responses. Participants who reacted positively to the app noted that it could be improved by the inclusion of a more welcoming color scheme and by having an option for text to be read aloud to the user, which would allow for better focus on the exercises.

### 4.4 | Theme 4

#### 4.4.1 | Those Who Did Not Use the App Cited Not Being Prompted to Use It, and a Desire for More Active Treatment

One-third of the participants had used the app only minimally, or not at all, prior to the interview. Reasons for this varied from not being inclined or prompted to use it to not being technologically savvy:

I have not been using it, but I did go through it initially, and it seems very user friendly. Also, I don’t tend to do a lot of digital technological things very much because I’m more old-fashioned, so I think that might have something to do with it, but I prefer paper and pen as opposed to looking at an actual app and utilizing that on a daily basis. I think that’s a huge factor for me not using on a daily basis….

This participant further described liking the features and content of the app, but being unwilling to use an app as opposed to pen and paper. Other participants wanted routine reminders to encourage app usage. Another participant who had used the app minimally noted that he became angry when the app identified problems without giving him access to more direct treatment:

To be honest with you, I’ve only been on it, like, twice…. like the avoiding triggers, that was for, I liked that. … when I get angry. It just asks you questions like, what’s your level of angriness and, you know, you answer it and then you know what I mean?… Maybe your stress level’s a little too high, you wanna talk to somebody.

These participants who minimally used the app also had negative reactions to the “inspiring” imagery and quotes, noting that seeing beautiful images of far-away places were frustrating as they reinforced the fact that they were not able to go to such places at the moment. Inspiring quotes were similarly not viewed as helpful, with this participant indicating a desire to use only active versus passive tools in Manage Symptoms:

So, let me pick one [a symptom] that I actually have, like unable to sleep. I’m going to put an eight next, and all I got was an inspiring quote that says, “I failed my way to success.” That’s not really helpful.

### 4.5 | Theme 5

### 4.5.1 | Journaling, Providing More Mechanisms for Participants to Shift Focus From Pain, and Being Able to Re-Name the App Were Suggestions to Tailor It to Benefit Acutely Injured Patients

When prompted to think about features of the app that could be improved upon to aid in recovery, and particularly with pain, some participants suggested the addition of features to help with emotional expression, such as a journaling section:

Yeah, like because I know for me like writing about my feelings like helps me also track my progress, and I know a lot of people who suffer from PTSD and it’s good to like—that’s another way to track your progress, plus like, just it’s cathartic to get your emotions out on paper, because then it’s easier to see they’re temporary, you know what I mean? I might feel this way right now, but it will pass and if I write it out I’ll like release some of the anxious energy and the stress from having to feel this way, so I would say, like, for me, a journaling section would be cool. Like maybe just, you know, something to like type in a few things how you’re feeling that day or like a check in for that day, so you can track your progress that way. Like, how are you feeling today? What were your stressors? Or like, you know, check off a box to maybe chart it better.

As participants have mentioned, a positive feature of PTSD Coach is its variety. In addition to journaling, incorporating features to aid in thought shifting/drawing attention away from the pain and ruminating thoughts would be helpful to manage pain:

…I mean, for me…the best thing that I do to help with my own pain and having to deal with it, is, is literally doing something that, else distracting, that makes me happy, because I can, um, tell you that talking — you know, not this discussion; it’s, it’s feasible and necessary — but talking about your pain continually does not, it’s not helpful. Um, when it’s not with the doctors, and not with, because you don’t, the getting into self-pity, um, you know, it’s necessary to be able to understand and reflect on your pain so that you can discuss it with your doctor, but then thinking about it constantly and feeling sorry for yourself is not useful because it’s not gonna get you out there and it’s not gonna help keep you motivated to, um, to live, survive, and keep doing things, and not roll over and say, I can’t take it anymore [Yawn], you know what I mean, um.

When prompted about the addition of tools that would help to manage pain and thoughts of pain, one participant noted that the app could assist with identifying and offering alternative approaches to medication in reference to pain management:

Yeah. I definitely, I would use that cuz sometimes, like — […] when you’re having a pain, all you want to do is hurry up and go get some medicine or, instead of, like, trying to cope with it because it’s not that serious but you could do other things instead of, like, medicating yourself and stuff like that, you know? So that’d be pretty cool. […. Coping strategies?] Yes, that’s – yeah. Definitely.

Lastly, one participant wanted the ability to re-name the app in their phone (customized for each user) to feel that they had “ownership” in their recovery process:

…Like, if you could rename it on your phone so that you could take ownership of it, I feel like that’s such a big thing in my experience, when I pull from my training with social work. Like, when you own it, you have some pride and, you know, there’s some accomplishment to be said about that.

### 4.6 | Theme 6

#### 4.6.1 | Most Participants Would Recommend PTSD Coach to Others

Despite that only 2/3^rds^ of participants used the app, 89% (*n* = 16) would recommend the app to others in their situation, primarily because they valued the resources offered, skills learned, and opportunity to monitor their symptoms through completion of regular self-assessments (e.g., PCL-5 scale and digital journaling). These quotes from two different participants demonstrate this point:

1. I would. I’d recommend it just to tell them, like, there’s different, like, symptoms that they may be experiencing that they can get, like, help from pretty much immediately before having to try something else.
2. Yes, because, as I said, it, it makes everything easier to keep track of, it…helps…give you ideas and exercises on how to calm your nerves, and it’s just very helpful.

Even those who did not think the app would “solve” issues found it to be valuable. Participants were introduced to new avenues of support, such as behavioral therapy (openness to treatment):

I would say yes, because, um, I think that anything related to psychology and getting anyone interested in helping themselves when they have trauma, is really important. Because even if the app doesn’t solve their issue — which I, I, it would be hard for the app itself to solve their issue — if it introduces them the, to the idea of reaching out to a therapist, um, to get themselves help, it’s, I, I think that’s wonderful. Um, because, um…because people shouldn’t suffer when they can go to someone and talk it out and get some therapy and get some help. And I think this app could, could be a fantastic resource for people, you know, gently being introduced to the idea that they can get help and they can get better, and they can be, you know, their lives can be improved.

Another participant said the app could benefit others, even though she preferred non technological ways to find support for herself:

I’m sure it could be helpful to some people and I’m sure it could benefit them, [but] I feel like there’s other avenues of support. Sometimes, you know [those other avenues are] being eliminated because of technology, and I just wish they would bring those back because there are people, other people like myself [who prefer in-person help].

## 5 | Discussion

This study represents the novel perspectives of acutely injured patients introduced to the PTSD Coach app during or shortly following an ED visit for a traumatic physical injury. Our proportion of injury mechanisms is similar to other studies, where most injuries presenting to the ED are due to falls, followed by general injuries, motor vehicle accidents, and interpersonal trauma (A et al. 2023). As the patients who provided feedback on the app were not yet eligible for a PTSD diagnosis, given the recency of the injury, we can only evaluate the impact of the app on post-injury distress or early stress reactions versus PTSD symptoms. Further, it is important to interpret our results with the caveat that recruitment was challenging, and we were not able to interview most of the participants who enrolled in the study; additionally, these results primary reflect the perspectives of women versus men, given the low number of men included in our sample. During interviews conducted within 3-months post-injury, participants described significant pain and emotional distress that contributed to logistical, informational, and existential challenges during their path to recovery. Specifically, many experienced disruptions to routines, transportation barriers and consequent financial and familial worries, and stressors associated with navigating disability-related paperwork and health insurance systems. Injury and pain-related problems also contributed to a lack of independence. It was difficult for participants to control pain-related symptoms at home due to challenges associated with navigating the healthcare system and a lack of knowledge about evidence-based resources to help manage a pain flare, prompting the use of independent internet searches for immediate pain-reduction techniques.

Consistent with positive feedback from Veterans and Civilians who used PTSD Coach to manage established PTSS, our sample of acutely injured patients endorsed the app as being a helpful resource to manage post-injury stress-related needs. Specifically, they enjoyed access to varied resources and tools to identify and cope with triggers and help manage and track PTSS. Patients also discussed how the app increased awareness of their symptoms and helped them implement proactive coping methods in attempts to deter negative outcomes (or at least manage expectations). Relatedly, they acknowledged the potential for PTSD Coach to bridge the gap between the emergence of symptoms and formal mental health services. This aspect is notable given that few people with PTSD are aware of symptoms, and only a small percentage seek treatment after years to decades have passed (see Smith et al. 2020).

Further, for patients with prior mental health treatment, the app may have served as a reminder to use tools for self-management that were helpful in the past. It is also notable that over a quarter of participants were actively seeking mental health therapy or counseling; although we did not probe for reasons, the willingness to seek therapy alone may have increased the likelihood to enroll in this study and to actually use the app. It cannot be ruled out that positive attitudes towards the app or perceived benefits gained from the app may be at least partially attributable to other forms of therapy versus content of the app alone. Albeit PTSD from a prior trauma can increase the risk of PTSD following a subsequent trauma (Breslau et al. 1999), and it may be particularly helpful for PTSD Coach to boost the impact of or serve as a reminder of skills learned in prior therapy. To that end, an appeal of low-risk digital health interventions like PTSD Coach is that they have the potential to be used to increase access to and connect patients to evidence-based care (Price et al. 2014), potentially reducing downstream healthcare costs (Gentili et al. 2022).

**FIGURE 2 |:**
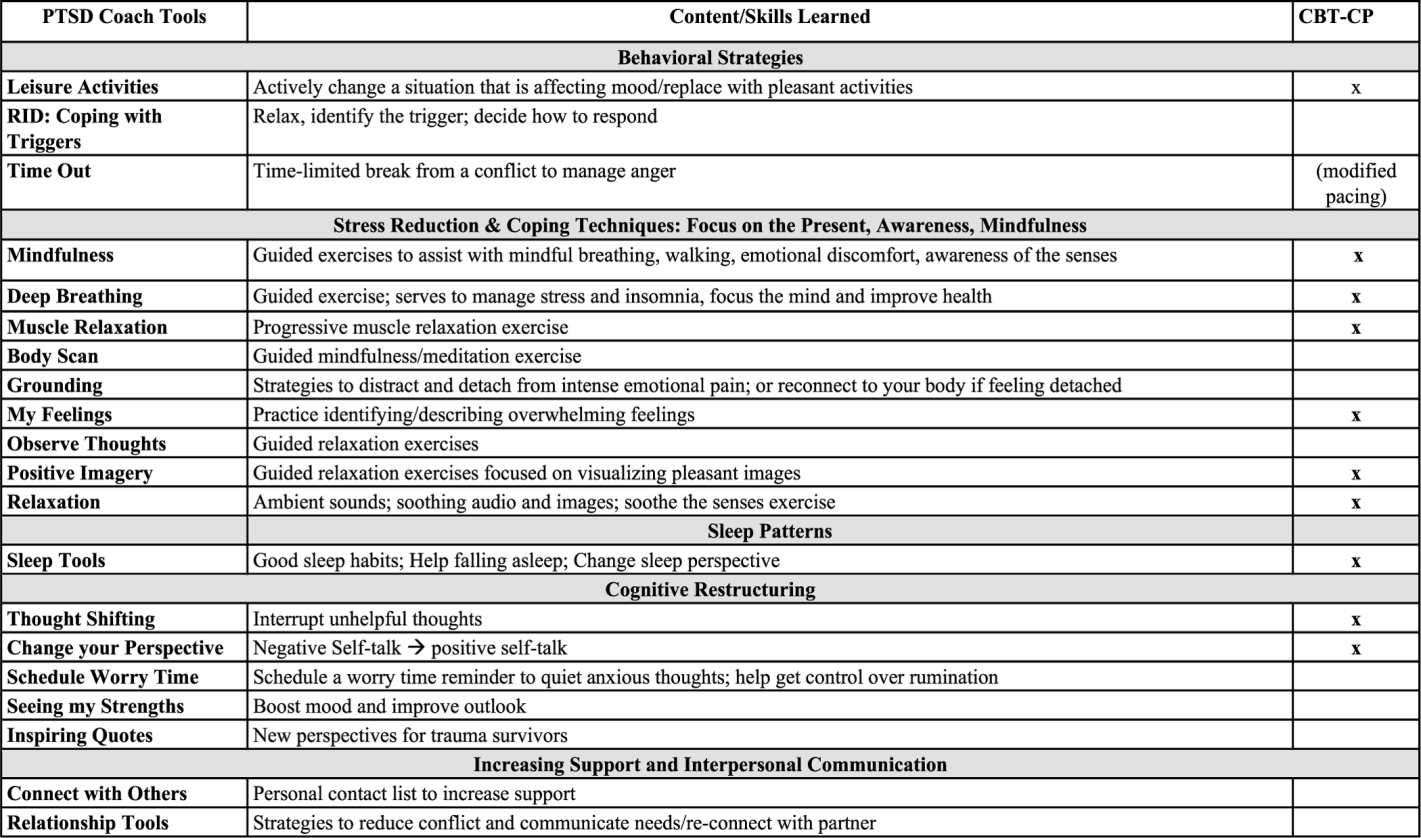
Tools in the PTSD Coach manage symptoms module, the content and type of skills that they are designed to facilitate, and the overlap of skills with CT-CP.

Notably, at least one patient viewed PTSD Coach as a pain-management app, suggesting that the tools within the manage symptom module may address elements of fear-avoidance that are critical to maintaining symptoms of both PTSS and pain (Bosco et al. 2013): these tools include cognitive restructuring to reshape and redirect maladaptive pain cognitions and mindfulness and relaxation to address heightened arousal central to both conditions (Liedl et al. 2010) (See Figure 2). Relatedly, patients acknowledged tools such as “time-out” that were beneficial to disrupt the negative cycle of thoughts that serve to maintain anxiety, particularly regarding uncertainty of recovery time and worries that pain will persist. These features central to PC, which serves to maintain pain, are critical to address when facilitating recovery. Suggestions to optimize PTSD Coach for use after injury included journaling to increase emotional expression and recognition that emotions are temporary (digital journaling is now available upon download of the most recent version of the PTSD Coach app), and additional strategies for distraction and/or to stop the cycle of pain rumination. To this end, PTSD Coach may be optimized by adding an adaptive coping checklist for pain, similar to one included in CBT-CP, to remind patients that pain flares are temporary. Yet, even without these optimizations, most participants stated they would recommend PTSD Coach to others. Moving forward, identifying the specific features that support post-injury recovery may serve to maximize overall effectiveness and engagement among this unique sample (Hallenbeck et al. 2022).

Participants perceived that PTSD Coach can increase self-awareness of symptoms, improve self-efficacy to manage acute distress, assist with symptom monitoring, and facilitate appropriate treatment seeking if needed outside of formal therapy (Corthésy-Blondin et al. 2023) (critical steps in recovery Kuhn et al. 2017). As such, this data suggests that PTSD Coach may fill a critical gap for recently injured patients in acute medical settings: despite widespread support for psychological approaches to improve post-injury health outcomes (Hsu et al. 2019; Pacella-LaBarbara et al. 2023a), there are no standard resources for patients, particularly for those with mild-to-moderate injury without access to resources provided during hospital admission, and for underserved patients with little connection to healthcare outside of their ED visit. It is also important to note that using the PTSD Coach app prior to 1-month post-injury, before a PTSD diagnosis could be prescribed, would be for the purpose of managing or preventing PTSD symptoms. To that end, modifying the name of the app for this specific population may serve to more accurately reflect its purpose as a prevention toolkit among acutely injured patients.

On the other hand, the app did not address post-injury logistical or informational challenges that tended to be structural in nature (e.g., navigating insurance and disability paperwork; length of time to see a physician); the addition of links with the app to established resources, such as application instructions for short-term disability, may help patients navigate these issues and reduce the need for independent time-consuming internet searches. Further, approximately one-third of ED patients either did not use or used PTSD Coach minimally prior to their interview; some patients preferred paper-and-pencil and/or face-to-face methods versus digital intervention for distress management, highlighting a resistance to using technology for mental health needs. For those willing to use digital interventions, several app additions may serve to improve engagement: longer exposure time to resources post-injury, reminder messages, options to enable text to be read aloud so users could listen to modules, the addition of more direct links to specific tools in the manage symptom section, and the option to customize imagery (e.g., make it brighter/more inviting) and the appearance of certain tools (e.g., quotes).

Although perceptions varied, some expressed dislike for certain tools such as inspiring imagery and quotes; future research is warranted regarding whether disabling specific tools within the app (based on the presence or absence of symptoms) may serve to improve personalization and potentially improve ease of navigating the tools within the app. Yet, this approach would require additional research into mechanisms of change, specifically in understanding which specific tools are necessary to produce a meaningful change in cognition and behavior. The app is already equipped with a feature that allows the ability to like and dislike certain app tools (which will either increase or decrease the likelihood of those tools appearing again), and the most recent version of PTSD Coach also displays users favorites on the home screen. Lastly, numerous participants expressed the desire for more personalization and interaction. Future work may also explore the potential for Generative Artificial Intelligence (AI) to supplement the app and provide participants with increased personalization within PTSD Coach (e.g., a generative AI chatbot may refer patients to sections of the app that may be particularly helpful based on symptomology). A recent randomized trial suggests that fully generative AI chatbots designed and supervised by mental health clinicians can reduce mental health symptoms(Heinz et al. 2025). Yet, it is critical for any digital tools to be co-designed with content experts to optimize the content and quality of psychoeducation, recommendations and resources, personalized to optimize outcomes, and thoroughly tested prior to widespread use (Torous et al. 2025).

Given the strained and unpredictable ED setting, recruiting ED patients into research studies is challenging (Price et al. 2020). Despite varied recruitment methods, only 41% of enrolled patients completed an interview. Notably, differences in recruitment method may impact willingness and readiness to use the app and to participate in research: both participants who voluntarily enrolled in this study via the research portal completed the interview. Conversely, all participants lost to follow up were recruited either via in-person methods or via the flyer in the ED. Insufficient rapport and participant motivations, two critical areas that impact participation in qualitative research (Negrin et al. 2022), may have led to high dropout rates: given that most patients did not have their schedules readily available during enrollment in the ED (e.g., cell phones were damaged or did not have a charge etc.), a staff member from the Qual EASE research team reached out to the patient within 1 week of enrollment to schedule the interview; such inconsistency between the staff member recruiting the patient and the staff member scheduling and conducting the interview may have led to poor rapport with the research team. We also approached patients with varied severity of injuries, most of which were mild to moderate; even among those who qualified for the study, patients may not have felt significant emotional distress from their injury and motivations to engage with the app or interview were lacking.

In general, the problem of low uptake for digital mental health interventions is widespread, in part due to uncertainty about the usefulness of these interventions and/or ease of usability, and stigma surrounding mental health (Aaron et al. 2024; Jardine et al. 2024). Further, study enrollment was minimally burdensome in the ED, but engaging in a one-on-one interview requires effort, time and willingness to discuss feelings and experiences with the app, all of which may contribute to dropout. Indeed, common barriers to seeking mental health treatment following injury include lack of time, knowledge, or resources, trauma-specific barriers (e.g., concerns about re-experiencing or avoidance symptoms of PTSD); and postinjury physical limitations(Kantor et al. 2017; Kazdin 2017; Manser et al. 2018).

Our sample also primarily reflects perspectives of women, as only four men completed an interview, one of whom used the app as directed. In general, men display more negative attitudes towards mental health treatment than women (e.g., less likely to acknowledge the helpfulness of psychotherapy (Pattyn et al. 2015), and men display more stigma in response to mental health treatment and treatment seeking than women (Fox et al. 2015). These findings are also consistent with studies that suggest men are more likely the women to avoid mobile health apps for mental health, and with an association between mental health and femininity: men anticipate that others perceive them as more feminine when engaged with digital mental health apps and are therefore less likely than women to use them (Lee and Trudel 2025). Despite the privacy, anonymity, and self-help features provided via mental health apps, additional work is needed to identify how to overcome significant barriers preventing mental health app engagement among high-risk men (Lee and Trudel 2025). Further, to better identify patients who may benefit from psychosocial support resources after injury, it may be helpful to incorporate the stage of change model to examine associations between theoretical behavior change mechanisms and the use of the PTSD Coach app (Crookston et al. 2017). Alternatively, PTSD Coach may be a particularly valuable resource for women, as they are more vulnerable to both PTSS and CP.

Additional study limitations include a lack of access to medical records to contextualize injury details and confirm discharge status following ED treatment: consequently, participants in our sample suffered from injuries ranging from mild to severe. Further, we did not monitor actual app usage given the qualitative nature of this study, and there was a short time where participants were asked to use the app (at least 1-week prior to the interview) despite that symptom changes in stress and pain may occur frequently over the course of the acute injury period (3-months post-injury). Yet, we purposely aimed to speak with patients shortly after injury to learn about their needs during a stressful, fraught time. Moving forward, future studies examining usage data in relation to symptom severity over the course least 3 months post-injury may be necessary to understand patterns of symptom improvement potentially attributable to resources included in the app. Relatedly, although it was beyond the scope of this qualitative analysis to determine mechanisms of action for PTSD Coach to improve recovery after injury, future research regarding coping styles and coping self-efficacy should be evaluated as modifiable mechanisms that the app targets (Hensler et al. 2022); additionally, EMA or diaries could be incorporated to determine the factors or triggers that prompt participants to use the app.

Future work is warranted to determine qualitative trends across age, gender, and injury type, as our sample size was designed to achieve thematic saturation regarding whether PTSD Coach app could be useful in a novel population; further work is also needed to determine the potential role for PTSD Coach to serve as an intervention to disrupt the formation of maladaptive behaviors that become habitual and impair recovery. Notably, the tools within the app may broadly serve to improve mental health and well-being (e.g., relationships with others; coping with daily stressors) (Corthésy-Blondin et al. 2023) rather than modify PTSS specifically; as such, PTSD Coach may be uniquely positioned to disrupt the dysfunctional thought and behavior patterns among those with subthreshold PTSS.

In sum, this study is the first to qualitatively examine perspectives on the app after acute injury; as such, this data sheds light on the needs and challenges of acutely injured patients and how the PTSD Coach app may fill an unmet resource gap. Beyond the presence of significant physical and emotional pain, isolation and loneliness further served as primary barriers to post-injury functioning. Participants had favorable feedback regarding the app’s ability to help manage and increase awareness of symptoms, cope with distressing reminders of the trauma, and to facilitate connections to mental health treatment. PTSD Coach was initially designed to address established PTSS among veterans, yet it has a promising impact on PC (Pacella-LaBarbara et al. 2023b) and PTSS among vulnerable populations (Bröcker et al. 2023); with optimization to increase engagement among acutely injured patients, it may further serve to help injured patients self-manage acute distress (Pacella-LaBarbara et al. 2020) and psychological symptoms that may impede physical and emotional recovery (Pacella-LaBarbara et al. 2023b). Although future work is necessary to optimize the app and increase uptake and engagement after injury, PTSD Coach may be an ideal candidate to support the development of and reinforce healthy thoughts, behaviors, and distress management skills to mitigate the psychological processes that magnify post-injury pain.

## Supplementary Material

Interview Script

Supporting Information

Additional supporting information can be found online in the Supporting Information section.

J clin psych interview script.

## Figures and Tables

**FIGURE 1 |:**
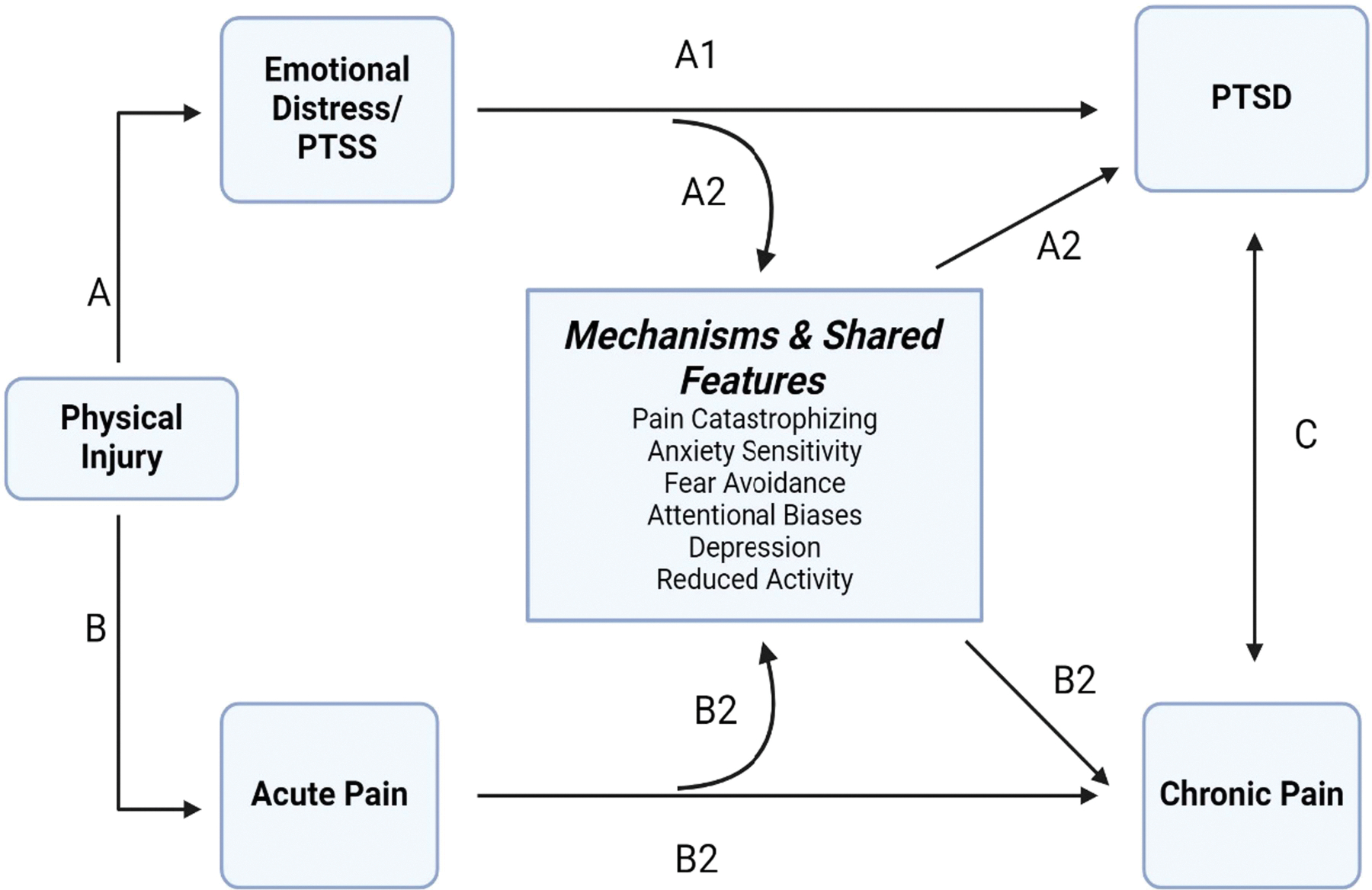
Path A: Injury triggers acute emotional distress responses including fear and initial PTSD Symptoms (PTSS), which can directly contribute to the development of PTSD at 1-month post-injury (A1), and indirectly contribute to chronic pain via pain-related processes such as pain catastrophizing (e.g., rumination, magnification, and helplessness) (A2). Such appraisals may continue to fuel fear and interpretations of pain-related sensations as threatening, prompting a cyclical process of fear, avoidance, and hypervigilance to such sensations which contribute to depression, reduced activity and ultimately chronic pain (A2). Path B: Injury also triggers acute pain sensations which can lead directly to the development of chronic pain (B1) or indirectly through catastrophic appraisals such as pain catastrophizing (B2). Path C: Both PTSS and pain-related symptoms are highly comorbid and share common cognitive, affective, and behavioral features (e.g., fear, avoidance; attentional biases, depression and reduce activity; anxiety sensitivity); these factors can contribute to the development of and/or maintenance of both PTSD and chronic pain (C).

## Data Availability

The authors have nothing to report.
