# Supplementary material for: A Qualitative Exploration of Post‐Injury Challenges and the Potential Role of the PTSD Coach Mobile Application to Improve Recovery Among Acutely Injured Patients
Source: J Clin Psychol. 2026 Jan 6;82(4):521–34. doi: 10.1002/jclp.70085 (PMC12958468; doi:10.1002/jclp.70085)
Supplement: Supplementary file 1 — J clin psych interview script. [file JCLP-82-521-s001.docx]

When you think about your injury, what did you find most challenging with regards to pain? Probes if needed: Pain at the moment of injury? Pain management during recovery?

How did you manage this?

What do you think could have helped you manage/reduce/overcome this?

(Before moving on): Did you have any other challenges related to pain that haven’t come up yet?

When you think about your injury, what did you find most challenging with regards to stress? Probes if needed: Stress at the moment of injury? Stress during recovery?

How did you manage this?

What do you think could have helped you manage/reduce/overcome this?

(Before moving on): Did you have any other challenges related to stress that haven’t come up yet?

When you think about your injury, what did you find most challenging with regards to recovery?

How did you manage this?

What do you think could have helped you manage/reduce/overcome this?

(Before moving on): Did you have any other challenges related to recovery that haven’t come up yet?

Now, I’d like to shift to talking about the app that you downloaded last week – (NAME OF APP HERE). Tell me about your general thoughts on the app.

Did you use it at all over the last week? If so, how?

What did you like, dislike about the app? Which modules (Learn about PTSD, Track Symptoms, Manage Symptoms, and Find Support) did you like or dislike?

Thinking back to our earlier discussion about pain, stress, and recovery: What features of the app could we add to help you with your pain, in particular?

There are currently no built-in features that assist you with learning about injury-related pain, tracking and managing pain, or that provide you with non-Veteran resources. Do you think you would use these features if we were to add them in? What would you like to see? What kind of tools do you think would help you manage pain and thoughts about pain?

What are your thoughts on receiving weekly text messages to track how you are handling pain and trauma from the injury, then getting personalized feedback about how to use the app based on your response?

What do you think would be the best name for a version of the app that would include pain features?

Would something like, “Recovery Coach” make it more appealing to use?

What could we do to make people like you more likely to use this app?

Are there any other specific changes you would make to the app? If yes, what? PROMPT: appearance; ease of use; wording; structure

Would you recommend the app to other patients? If yes, why? If no, why not?

That was my last question. Is there anything else that you think we should know? Is there anything you expected us to ask about that we didn’t?

Thank you!
